# TIL Therapy in Lung Cancer: Current Progress and Perspectives

**DOI:** 10.1002/advs.202409356

**Published:** 2024-10-18

**Authors:** Weilei Hu, Yifei Bian, Hongbin Ji

**Affiliations:** ^1^ Key Laboratory of Systems Health Science of Zhejiang Province School of Life Science Hangzhou Institute for Advanced Study University of Chinese Academy of Sciences Hangzhou 310024 China; ^2^ Key Laboratory of Multi‐Cell Systems Shanghai Institute of Biochemistry and Cell Biology Center for Excellence in Molecular Cell Science Chinese Academy of Sciences Shanghai 200031 China; ^3^ University of Chinese Academy of Sciences Beijing 100049 China; ^4^ School of Life Science and Technology Shanghai Tech University Shanghai 200120 China

**Keywords:** Adoptive cell transfer (ACT), Lung cancer, Non‐small cell lung cancer (NSCLC), Tumor‐infiltrating lymphocytes (TILs), Tumor‐reactive T cells

## Abstract

Lung cancer remains the most prevalent malignant tumor worldwide and is the leading cause of cancer‐related mortality. Although immune checkpoint blockade has revolutionized the treatment of advanced lung cancer, many patients still do not respond well, often due to the lack of functional T cell infiltration. Adoptive cell therapy (ACT) using expanded immune cells has emerged as an important therapeutic modality. Tumor‐infiltrating lymphocytes (TIL) therapy is one form of ACT involving the administration of expanded and activated autologous T cells derived from surgically resected cancer tissues and reinfusion into patients and holds great therapeutic potential for lung cancer. In this review, TIL therapy is introduced and its suitability for lung cancer is discussed. Then its historical and clinical developments are summarized, and the methods developed up‐to‐date to identify tumor‐recognizing TILs and optimize TIL composition. Some perspectives toward future TIL therapy for lung cancer are also provided.

## Graphical abstract

Unveiling the promise of Tumor‐Infiltrating Lymphocytes (TIL) therapy for lung cancer treatment. This review introduces why TIL therapy is suitable for lung cancer, delves into its historical and clinical developments, and discusses methods for identifying tumor‐recognizing TILs and optimizing TIL composition. It also provides some perspectives toward future TIL therapy for lung cancer.

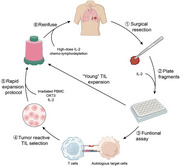

## 1 Introduction

Lung cancer is one of the most common malignant cancers worldwide and the primary cause of cancer‐related mortality.^[^ ^1^ ^]^ Non‐small cell lung cancer (NSCLC) makes up ≈85% of all primary lung cancers, with adenocarcinoma (40%) and squamous cell carcinoma (30%) as the main subtypes.^[^ ^2^ ^]^ The emergence of immune checkpoint blockade (ICB) has shifted the paradigm of lung cancer treatments, extending the survival of patients with advanced‐stage NSCLC.^[^ ^3^, ^4^ ^]^ However, overall survival and cure rates still remain low, with a 5‐year survival rate of only 26%.^[^ ^5^ ^]^ Many patients fail to respond to immune checkpoint inhibitors (ICIs), partially due to a lack of functional T cell infiltration. This challenge can potentially be addressed through adoptive cell transfer (ACT), which involves treating patients with immune effectors propagated ex vivo and then administered back. Currently, different forms of T cell‐based ACT such as chimeric antigen receptor (CAR)‐T therapy, T cell receptor (TCR)‐T therapy, and tumor‐infiltrating lymphocyte (TIL) therapy are being explored for the treatments of lung cancer, each at various stages of clinical development. TIL therapy refers to expanding TILs from resected/biopsy tumors ex vivo and reinfusing them back into the patients. The infused TILs are characterized by a collection of polyclonal T cells infiltrated into the tumor, offering distinct advantages such as polyclonality, tumor‐targeting capabilities, and low off‐target toxicity. Furthermore, the US Food and Drug Administration (FDA) has approved the first TIL therapy, Iovance's lifileucel (Amtagvi), for ICI or BRAF inhibitor‐treated patients with unresectable/metastatic melanoma.^[^ ^6^ ^]^ This approval has significantly boosted hopes for the potential of TIL therapy in treating lung cancer. In this review, we first introduce TIL therapy and its unique features and then discuss why TIL therapy is suitable for lung cancer. We then present the history of TIL therapy and its clinical development in lung cancer. We also detail the different methods developed to identify tumor‐recognizing TILs and optimize TIL composition. Last, we present some perspectives toward future TIL therapy for lung cancer.

## 2 Characteristics of TIL Therapy

TIL therapy, one of the earliest developed ACTs holds the distinction of being the first ACT to receive approval from the US FDA for solid tumor treatment.^[^ ^6^ ^]^ This innovative therapy harnesses the patient's natural tumor‐infiltrating T cells, which are isolated from the tumor, expanded in large numbers, and then re‐infused into the same patient to boost the immune response against cancer.

While CAR‐T and TCR‐T therapies are two other widely recognized forms of tumor‐specific T cell‐based ACT, they rely on the genetic modification of T cells derived from the patient's peripheral blood to enhance their tumor‐fighting capabilities. CAR‐T cells, in particular, eliminate target cells through antigen–antibody recognition and have proven especially effective against well‐defined surface antigens like CD19 in hematologic malignancies. Several CD19‐targeted CAR‐T therapies have been FDA‐approved for treating B cell malignancies.^[^ ^7^ ^]^ However, due to the complexity of solid tumor antigens, designing suitable antigen receptors is quite challenging, which limits CAR‐T therapy to the treatment of blood cancers. Furthermore, the high tumor burden of solid tumors requires the infusion of large numbers of immune cells, increasing the risk of “on‐target, off‐tumor (OTOT)” toxicity against non‐malignant tissues bearing target antigens. Additionally, the in vivo rapid expansion of CAR‐T cells and target cell killing could result in cytokine release syndrome (CRS), a serious side effect. These safety concerns significantly hinder the use of CAR‐T therapy in treating solid tumors.^[^ ^8^ ^]^

TCR‐T cells are genetically engineered to express specific TCRs targeting intracellular antigens presented on MHC molecules, allowing TCR‐T therapy to attack a broader range of tumor‐specific antigens and showing promising potential in solid tumors. TCRs against tumor‐specific antigens are typically screened from TILs or peripheral blood mononuclear cells (PBMCs) and then introduced into T cells via retroviral transduction.^[^ ^9^ ^]^ Lately, the US FDA approved afami‐cel, targeting HLA‐A*02‐MAGE‐A4, for the treatment of unresectable or metastatic synovial sarcoma.^[^ ^10^ ^]^ Like CAR‐T therapy, TCR‐T therapy also faces challenges such as CRS and OTOT toxicities, and cross‐reactivity may occur when cross‐reactive epitopes in normal tissues are present.^[^ ^11^ ^]^

In contrast, TIL therapy offers multiple advantages in treating solid tumors. TIL‐ACT comprises diverse T cell clones that recognize multiple tumor antigens, effectively addressing the inherent heterogeneity of solid tumors. Many TILs target neoantigens, bypassing central tolerance and reducing the risk of off‐target toxicity in non‐tumor tissues.^[^ ^12^ ^]^ Importantly, there have been no reports of OTOT or CRS toxicities associated with TIL therapy. Moreover, TILs, derived directly from tumor tissue, are naturally equipped to infiltrate the tumor. Unlike CAR‐T cells, which may face a mismatch between chemokine receptors and the chemokines secreted by solid tumors, TILs often consist of effector memory T cells expressing chemokine receptors like CX3CR1^[^ ^13^ ^]^ and CCR4,^[^ ^14^ ^]^ enhancing their ability to penetrate solid tumors.^[^ ^15^, ^16^ ^]^ These attributes position TIL therapy as a prospective treatment option for solid malignancies, including lung cancer.

## 3 The Immunogenicity of Lung Cancer

NSCLC is considered an immunogenic tumor with a relatively high tumor mutation burden (TMB), especially for those from smokers.^[^ ^17^ ^]^ Although genetic analysis has shown considerable heterogeneity among lung cancer patients, most tumors exhibit many common or “public” mutations. For lung adenocarcinoma, the most common mutations occur in *TP53, KRAS, EGFR, BRAF, NF1, KEAP1, STK11*, and *PIK3CA*.^[^ ^18^ ^]^ In contrast, lung squamous cell carcinoma is closely associated with smoking, and characterized by high genomic complexity, with commonly mutated genes including *TP53, PIK3CA, CDKN2A, PTEN, KEAP1, NFE2L2, MLL2*, and *RB1*.^[^ ^19^, ^20^ ^]^ In addition, lung cancers express unmutated tumor antigens, such as MAGE‐A3, a cancer‐testis antigen predominantly found in ≈30–50% of NSCLC patients.^[^ ^21^ ^]^ The generation of non‐mutated tumor‐associated antigens or mutated neoantigens forms the basis of lung cancer immunogenicity, which directly influences TILs in NSCLC. TILs contain a diverse group of lymphocytes, dominantly T cells with few B cells, and/or natural killer cells. They are present in tumor tissues and serve as an indicator of the host immune system's defense against cancer cells, representing the dynamic process of “cancer immune editing”.^[^ ^22^ ^]^ Several studies have shown an association between TILs and patient outcomes in NSCLC.^[^ ^23^, ^24^ ^]^ Also, an increased number of tumor‐infiltrating CD8+ T cells in lung cancer patients serves as a positive prognostic indicator,^[^ ^25^ ^]^ and a reliable predictor for assessing the response to ICB therapy.^[^ ^26^ ^]^ Mechanistically, these tumor‐infiltrating T cells, containing diverse TCR clones, patrol within lung cancer tissues, scanning for peptide‐major histocompatibility complex (pMHC) complexes on cancer cells or specialized antigen‐presenting cells (APCs) that can specifically activate their TCRs. T cells will stop migrating once encountering matched pMHC.^[^ ^27^ ^]^ The resistance, recurrence, and metastasis of lung cancer often arise due to the emergence of tumor subclones with accumulated mutations.^[^ ^28^ ^]^ If T cells can recognize these tumor subclones, they can theoretically mediate cancer cell killing and thus result in tumor regression.^[^ ^28^ ^]^ These mechanisms underpin the adoptive transfer of TILs with diverse TCR repertoires in vivo, establishing the promise of TIL therapy as a unique anticancer treatment for highly heterogeneous tumors such as lung cancer.^[^ ^11^, ^12^ ^]^

## 4 Historical Advances of TIL Therapy

### 4.1 The Origin of TIL Therapy

TIL therapy has evolved through decades of research and clinical trials. Dr. Steven Rosenberg and his colleagues played a crucial role in pioneering adoptive cell therapies. They initially isolated lymphocytes from cancer patients' blood and utilized interleukin‐2 (IL‐2) as a critical immune cell activator, laying the foundation for long‐term T cell culture.^[^ ^29^ ^]^ They developed recombinant IL‐2 in 1983, which facilitated large‐scale production of IL‐2 and boosted the clinical application of T cell‐based therapy.^[^ ^30^ ^]^ In 1985, a clinical trial involving autologous ACT of lymphokine‐activated killer (LAK) cells showcased the safety and feasibility of such therapy, with one patient undergoing complete remission.^[^ ^31^ ^]^ This phenomenon suggests that the key to the success of ACT is to identify immune cells capable of specifically targeting cancer. Therefore, Steven Rosenberg et al. initiated the tests to determine if TIL from freshly resected tumors could thrive in IL‐2 over extended periods and effectively recognize tumors. In a murine experiment, they demonstrated the advantages of TIL over LAK in mice with micrometastases from various tumor types undergoing cell infusion, including better fold‐expansion, enhanced tumor reactivity, and longer response duration.^[^ ^32^ ^]^ In the follow‐up experiment, Steven Rosenberg et al. explored TIL therapy in mice with large lung and liver metastases unresponsive to LAK therapy.^[^ ^33^ ^]^ The combination of TIL and cyclophosphamide effectively eradicated metastatic lesions in the liver and lung, and adding IL‐2 further enhanced the effect. With TIL plus IL‐2 in combination with cyclophosphamide, all advanced liver metastases and up to 50% of lung metastases in mice bearing MC‐38 colon adenocarcinoma were cured. This preclinical investigation provides the rationale for TIL therapy in treating human cancer. In 1987, they further conducted a pilot clinical study that demonstrated the safety and feasibility of autologous TIL adoptive transfer, with improvements observed through dose escalation of TIL quantity, IL‐2 in patients with lymphodepletion preconditioning using cyclophosphamide treatment.^[^ ^34^ ^]^ They reported the first clinical trial of TIL therapy for metastatic melanoma in 1988^[^ ^35^ ^]^ and further summarized the clinical findings in 1994.^[^ ^36^ ^]^ In these clinical studies, TIL cultures were produced in 6000 IU ml^−1^ IL‐2 until an average of over 1 × 10^11^ lymphocytes was achieved. Subsequently, a total of 86 patients were administrated with autologous TILs regardless of tumor recognition. These patients also received high dose intravenous IL‐2 of 720 000 IU kg^−1^ every 8 h, with 57 of them additionally receiving a single dose of 25 mg k^−1^g cyclophosphamide before infusion of TILs and IL‐2. Surprisingly, this trial showed a promising  objective response rate  (ORR) at 34%. These studies establish TIL therapy as a potential therapeutic option and pave the way for institutions worldwide to explore its potential.

### 4.2 The Optimization of TIL Production Process

The success of TIL therapy requires a large number of expanded TILs. Advances in T cell biology and culture techniques have enabled the rational refinement of TIL production methods, leading to the development of the rapid expansion period (REP) process, which now plays a pivotal role in TIL therapy. REP involves culturing TILs in a medium supplemented with IL‐2, OKT3 (α‐CD3ε monoclonal antibody) first, and then allogeneic feeder cells for an additional 14 days, which enables TIL expansion for more than 1000 folds.^[^ ^37^ ^]^ The CD3 subunit complexes (*γ, δ, ε, ζ*) perform key roles in transducing epitope‐recognizing signals into the cytoplasm and modulating TCR complex expression. Stimulating the TCR‐CD3 complex by adding OKT3 during the REP can enhance T cell fold‐expansion and long‐term culture.^[^ ^38^ ^]^ It also generates T cells with stronger persistence and reduced IL‐2 dependency.^[^ ^39^ ^]^ Another critical element of REP is the use of allogeneic feeder cells. Richard L. Kradin et al. used irradiated PBMCs as feeder cells to attain a large number of TILs for infusion.^[^ ^40^ ^]^ Irradiated PBMCs preserve their co‐stimulatory capacity without competitive proliferation. Steven Rosenberg et al. have recently adopted REP in the clinical trials of TIL therapy.^[^ ^37^ ^]^ Of course, a better understanding of REP and detailed culture condition and expansion mechanisms will certainly help the future development of optimal TIL therapy.

### 4.3 The Establishment of TIL Therapy Regimen

**Figure 1:**
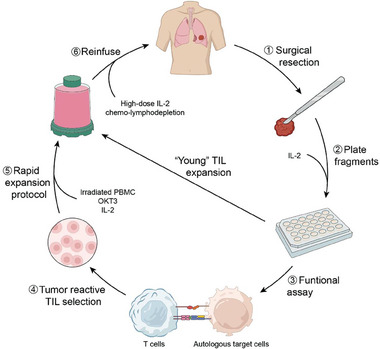
TIL Production and Administration. Step 1: the tumor specimen is resected and divided into multiple fragments. Step 2: individual tumor fragments are grown in a T cell medium containing high‐dose IL‐2 at 6000 IU ml^−1^. Within 2 to 3 weeks, TILs proliferate and kill tumor cells, generating pure TIL cultures. Step 3: these initially expanded TILs are tested for tumor reactivity in co‐culture assays. Step 4: tumor‐reactive TIL mini‐cultures are selected. Step 5: selected TILs are expanded to large cell numbers during the Rapid Expansion Protocol (REP) with irradiated peripheral blood mononuclear cells (PBMCs), OKT3 antibody, and IL‐2 at 3000 IU ml^−1^. Step 6: Ex vivo expanded TILs are reinfused into the patient accompanied with IL‐2 (720,000 IU kg^−1^). Non‐myeloablative lymphodepletion (LD) is administered prior to TIL infusion, typically involving the administration of 60 mg kg^−1^ cyclophosphamide for 2 days and 25 mg m^−^^2^ fludarabine for 5 days. To shorten the ex vivo time, the “young TIL” approach involves expanding TIL mini‐cultures directly by REP without selection. Up to 1 × 1011 lymphocytes can be obtained for infusion into patients pretreated with chemo‐lymphodepletion.

TIL production involves an appropriate manufacturing practice‐compliant ex vivo expansion process based on surgically excised tumor tissues. Ideally, tumors are obtained through minimally invasive procedures, for example, biopsies from subcutaneous tumors or lymph node metastases.^[^ ^41^, ^42^ ^]^ The standard approach of TIL production for clinical trials was settled in 2003 (**Figure** **1**).^[^ ^37^, ^43^ ^]^ Tumor tissues are processed through mechanical fragmentation or enzymatic digestion. Typically, multiple fragments from a single tumor are cultured independently in wells containing high‐dose IL‐2. Over a period of 2–3 weeks, T lymphocytes migrate into the culture medium and outcompete other cells, forming the mini‐culture of TIL. In the standard approach, the cytotoxicity of TIL mini‐cultures is assessed by co‐culturing them with autologous tumor cells or peptide/minigenes pulsed APCs. Only individual cultures demonstrating high toxicity against target tumors are chosen for further expansion. This entire process typically takes 6–8 weeks.^[^ ^44^ ^]^ Nevertheless, TILs are prone to exhaustion after such a long in vitro cultivation and may not persist in patients. To address this problem, the “Young TIL” method was introduced, which rapidly expands TILs for infusion without tumor‐reactivity selection, cutting the total ex vivo time by nearly half (Figure 1).^[^ ^45^, ^46^ ^]^ Using this approach, ≈1 × 10^11^ lymphocytes can be harvested within 5–6 weeks culturing. Recent industrial efforts have further improved the production process, for example, Iovance company develops a streamlined 22‐day good manufacturing practice (GMP) procedure for TILs (lifileucel), known as the Gen‐2 process, which reduces the tumor fragment culture (Pre‐REP) to 11 days. This method yields ≈1 × 10^8^ bulk TILs, which are then subjected to an additional 11‐day REP culture.^[^ ^47^, ^48^ ^]^ Non‐myeloablative lymphodepletion is proceeded in patients before TIL infusion, which typically involves the administration of 60 mg k^−1^g cyclophosphamide over 2 days plus 25 mg m^−2^ fludarabine over 5 days. Such a pre‐condition chemotherapy regimen significantly enhances the effectiveness of TIL therapy through eliminating immune suppressive cells including regulatory T cells (Tregs) and myeloid‐derived suppressor cells in patients or limiting the competition of cytokines like IL‐7 or IL‐15.^[^ ^49^ ^]^ This is followed by the transfer of cells and high dose IL‐2 (720 000 IU kg^−1^) until tolerance is achieved.^[^ ^50^ ^]^

## 5 The Clinical Application of TIL Therapy in Lung Cancer

The accelerated approval granted by the US FDA to lifileucel (Amtagvi) signifies a great advancement in melanoma patient treatments. However, for lung cancer patients, the opportunity to receive such treatment still remains limited to clinical trials. The details of clinical trials regarding TIL therapy are outlined in **Table** **1** (Updated to July‐2024). Here, we focus on the clinical developments of TIL therapy in lung cancer.

**Table 1:** Clinical trials of TIL therapy in lung cancer (data from https://clinicaltrials.gov/, updated to July‐2024).

Trial identifier	Cancer type	Phases	Enrollment	Study status	Interventions	Locations	Sponsor
NCT00019084	Advanced Lung Cancer	II	NA	Completed	TILs + IL‐2 + mutant p53 peptide‐pulsed dendritic cell vaccine/ras peptide cancer vaccine + sargramostim	United States	National Cancer Institute
NCT04842812	Advanced Lung Cancer	I	40	Recruiting	TILs/CAR‐TILs	China	Second Affiliated Hospital of Guangzhou Medical University
NCT06107894	Advanced Lung Cancer	I	12	Not yet recruiting	TILs (NEOG‐100) + IL‐2 + NMA	South Korea	NeogenTC
NCT05366478	Advanced NSCLC	I	15	Recruiting	TILs (LM103) + IL‐2	China	Suzhou BlueHorse Therapeutics Co., Ltd.
NCT05393635	Advanced NSCLC	I	0	Withdrawn	TILs (ITIL‐168) + IL‐2 + Pembrolizumab	United States	Instil Bio
NCT05397093	Advanced NSCLC	I	51	Active not recruiting	TILs (ITIL‐306) + NMA	United States	Instil Bio
NCT05576077	Advanced NSCLC	I	60	Recruiting	TILs (TBio‐4101) + IL‐2 + NMA + radiation + Pembrolizumab	United States, Canada	Turnstone Biologics, Corp.
NCT03215810	Advanced NSCLC Metastatic NSCLC	I	20	Completed	TILs (LN‐145) + IL‐2 + NMA + Nivolumab	United States	H. Lee Moffitt Cancer Center and Research Institute
NCT05681780	EGFR, ALK, ROS1, or HER2‐Driven NSCLC	I/II	20	Recruiting	TILs + IL‐2 + NMA + Nivolumab	United States	H. Lee Moffitt Cancer Center and Research Institute
NCT05483491	Lung Cancer	I	42	Recruiting	KK‐LC‐1 TCR‐T cells + IL‐2 + NMA	United States	Christian Hinrichs
NCT05600933	Lung Cancer	NA	1200	Enrolling by invitation	NA	United States	National Cancer Institute
NCT05902520	Lung Cancer	I	18	Recruiting	CD8 CD39 CD103 TIL + IL‐2 + NMA	United States	AgonOx, Inc.
NCT06375187	Metastatic Lung Cancer Advanced Lung Cancer	I	18	Recruiting	TILs (GC203) + PD‐1 antibody	China	Shanghai Juncell Therapeutics
NCT06060613	Metastatic Lung Cancer Metastatic NSCLC	I/II	52	Recruiting	TILs (OBX‐115) + IL‐2 + NMA	United States	Obsidian Therapeutics, Inc.
NCT04614103	Metastatic NSCLC	II	170	Recruiting	TILs (LN‐145) + IL‐2 + NMA	United States, Canada, Germany, Netherlands, Switzerland	Iovance Biotherapeutics, Inc.
NCT06473961	Metastatic NSCLC	I	20	Not yet recruiting	TILs (GC101) + PD‐1 antibody	China	Shanghai Juncell Therapeutics
NCT05566223	Metastatic NSCLC Stage IV NSCLC	I/II	70	Not yet recruiting	CISH Inactivated TILs + IL‐2 + NMA	United States	Intima Bioscience, Inc.
NCT03419559	NSCLC	II	0	Withdrawn	TILs (LN‐145) + IL‐2 + NMA + Durvalumab	United States	Iovance Biotherapeutics, Inc.
NCT03645928	NSCLC	II	178	Recruiting	TIL (LN‐145) + IL‐2 + NMA + Pembrolizumab/ Ipilimumab/Nivolumab	United States, Canada, France, Germany, Greece, Spain, Switzerland, United Kingdom	Iovance Biotherapeutics, Inc.
NCT03903887	NSCLC	I/II	20	Unknown	anti‐PD1 antibody‐activated TILs + docetaxel and cisplatin regimen chemotherapy	China	Sun Yat‐sen University
NCT05573035	NSCLC	I	108	Recruiting	TILs (LYL845)	United States	Lyell Immunopharma, Inc.
NCT05878028	NSCLC	II	33	Recruiting	PD1 positive TILs + Tislelizumab + Docetaxel	China	Quanli Gao
NCT06235242	NSCLC	NA	20	Recruiting	TILs (GT201) + Teraplizumab	China	Grit Biotechnology
NCT06455917	NSCLC	II	30	Not yet recruiting	TILs + IL‐2 + NMA	Switzerland	University Hospital, Basel, Switzerland
NCT04919616	Recurrent NSCLC Refractory NSCLC	I	15	Not yet recruiting	TILs	China	Shanghai OriginCell Therapeutics Co., Ltd.
NCT05361174	Stage III NSCLC Stage IV NSCLC	I/II	53	Recruiting	TILs (IOV‐4001) + IL‐2 + NMA	United States	Iovance Biotherapeutics, Inc.

NSCLC: non‐small cell lung cancer; TIL: tumor infiltrating lymphocyte; CAR: chimeric antigen receptor; NMA: non‐myeloablative lymphodepletion; NA: not available.

The first trial assessing the efficacy of adoptive TIL therapy in the post‐operative treatments of Stage II, IIIa, or IIIb NSCLC was published in 1996.^[^ ^51^ ^]^ The study included 113 patients randomly assigned to standard chemoradiotherapy or adoptive TIL groups, with TIL showing no significant difference in mean survival for stage II patients but remarkably higher survival rates for stage III patients (22.4 months vs 8.9 months, *p *< 0.01).^[^ ^51^ ^]^ The number of transferred TILs ranged from 0.4—7 × 10^10^, accompanied by subcutaneous IL‐2 administration, without lymphodepletion preconditioning. Despite of limitations in the regimen, this study provides preliminary evidence supporting that TIL therapy can be successfully applied to treat advanced NSCLC as an adjuvant therapy.

Recently, an open‐label phase I trial (NCT03215810) evaluated the toxicity and preliminary efficacy of autologous TILs in conjunction with nivolumab for advanced NSCLC patients progressing on nivolumab monotherapy. Most patients (11/16) exhibited an initial response one month after infusion. Among 13 patients eligible for clinical evaluation, six exhibited radiographic responses, with two of them achieving complete responses that lasted over 1.5 years. One of the complete responders with an EGFR^ΔEx19^ mutation had infused TILs recognizing a somatic mutation (TMPRSS11F^S306L^) and several MAGE cancer‐testis antigens (MAGE‐A6, MAGE‐A4, MAGE‐A1) abnormally expressed in the tumor. Exploratory comparisons further indicate that complete or partial responders tend to have T cells recognizing tumor antigens. This suggests that TIL products can be administered safely and benefit individuals with advanced NSCLC resistant to PD‐1 therapy.^[^ ^52^ ^]^

IOV‐COM‐202 (NCT03645928) is an open‐label, prospective, multicenter, multicohort, phase 2 study assessing TIL therapy (Lifileucel) in patients with solid tumors. In the patients with ICI‐naïve metastatic NSCLC, the lifileucel plus pembrolizumab regimen resulted in an encouraging ORR at 42.1% (8/19), an even higher rate at 58.3% (4/8) for patients with EGFR wild‐type and PD‐L1‐negative. Some patients underwent durable and profound responses lasting 15.4 months and beyond, endorsing the use of this combination therapy throughout the disease course.^[^ ^53^ ^]^ Additionally, lifileucel as a monotherapy in ICI‐treated metastatic NSCLC patients reached an ORR at 21.4% (6/28).^[^ ^54^ ^]^ Among responders, two patients were PD‐L1 negative, among which one had an ongoing complete response at 26.3 months. Objective responses were also seen in patients with TMB‐low, STK11‐ and KEAP1‐mutated tumors, which were often considered resistant to immunotherapy in metastatic NSCLC. Notably, five responders exhibited deepening responses over time, with a sustained reduction of tumor lesions after initial evaluation. This suggests that a one‐time lifileucel monotherapy can produce durable and deepening responses in advanced NSCLC patients pretreated with ICI, endorsing more investigation.

## 6 The Parameters of TIL Products

TIL therapy represents a highly personalized treatment. The key parameters of TIL products include total cell count, CD8 T cell ratio, clonal diversity, proliferative capacity, and antitumor reactivity, which collectively determine the success of TIL therapy.

In a phase II clinical trial of TIL therapy for metastatic melanoma, the total count of transferred TILs emerged as a critical determinant of clinical responses, with the responders receiving nearly twice the average number of TILs compared to non‐responders. These findings underscore the importance of maximizing T cell infusion in TIL therapy to ensure more consistent clinical benefits.^[^ ^55^ ^]^

Higher CD8:CD4 ratios are associated with enhanced anti‐tumor reactivity.^[^ ^55^ ^]^ This is likely due to the existence of Treg cells which suppress the anti‐tumor responses. Interestingly, Chenwei Wang et al. identified that an EBV‐specific CD4 TCR‐T therapy can inhibit the growth of HLA‐DP5+ nasopharyngeal cancer in mice.^[^ ^56^ ^]^ Later study also shows that CD4+ T cells derived from bladder cancers demonstrate the capability to lyse autologous tumors.^[^ ^57^ ^]^ Several case reports indicate that transfusion of tumor‐reactive CD4+ T cells alone can induce significant anti‐tumor effects,^[^ ^44^, ^58^ ^]^ underscoring their importance in human cancer immunity. These transferred CD4+ T cells can facilitate tumor regression by aiding CD8+ T cells or exerting direct anti‐tumor effects through secretion of T helper 1 (Th1) cytokines like IFN‐γ and TNF‐α, or directly targeting MHC II‐expressing tumor cells via granzymes and perforins.^[^ ^59^, ^60^, ^61^ ^]^ Moreover, Bastian Kruse et al. found that a small subset of CD4+ cytotoxic T cells can eliminate MHC I‐deficient tumors that evade CD8+ T cell targeting.^[^ ^62^ ^]^ Notably, TIL products from various cancers, including lung cancer and melanoma, have shown a high proportion of CD4+ T cells, including CD4+ cytotoxic T cells.^[^ ^52^, ^63^, ^64^ ^]^ This phenomenon may be attributed to the crucial role of IL‐2 in the development of the cytotoxic program of CD4+ T cells though the Blimp‐1/granzyme B axis.^[^ ^62^, ^65^ ^]^ The impact of the CD8 T cells ratio on the efficacy of TIL therapy warrants reconsideration, and further exploration is urgently needed to determine the optimal ratio.

Previous studies have shown that increased telomere length in infused TILs and elevated expression of CD27 and CD28 correlate with improved clinical effectiveness and patient prognosis.^[^ ^66^, ^67^ ^]^ However, its predictive value still remains uncertain. Another study has shown that CD27− TILs with an effector phenotype achieve better outcomes compared to CD27+ TILs with a memory phenotype.^[^ ^55^ ^]^ In fact, the majority of tumor‐reactive T cells were found in a terminally differentiated state. Steven Rosenberg et al. conducted a phenotypic analysis of TIL infusion products from patients with complete responses to TIL therapy as well as non‐responders. They identified that a small number of tumor‐reactive T cells, at the stem‐like state with a CD39−CD69− phenotype, was correlated with durable remission.^[^ ^14^ ^]^ Therefore, investigating the phenotypic profiles of stem‐like memory T cells in cancer remains potentially important for enhancing TIL therapy efficacy.

A major strength of TIL populations lies in their capability to target multiple tumor antigens. In the phase I study targeting NSCLC patients (NCT03215810), both CD8 and CD4 T cells capable of recognizing cancer‐testis antigens and neoantigens were found in TIL infusion products, and these cells were strongly associated with objective clinical response.^[^ ^52^, ^68^ ^]^ This suggests that the greater the clonal diversity within TIL products, the more diverse the arsenal against tumor antigens, thereby increasing the likelihood of therapeutic response. Therefore, the key to determine the effectiveness of TIL therapy depends on how T cells are screened, how tumor antigen‐reactive T cells are re‐activated and propagated.

## 7 TIL Selection Based on its Reactivity against Tumor Antigens

**Figure 2:**
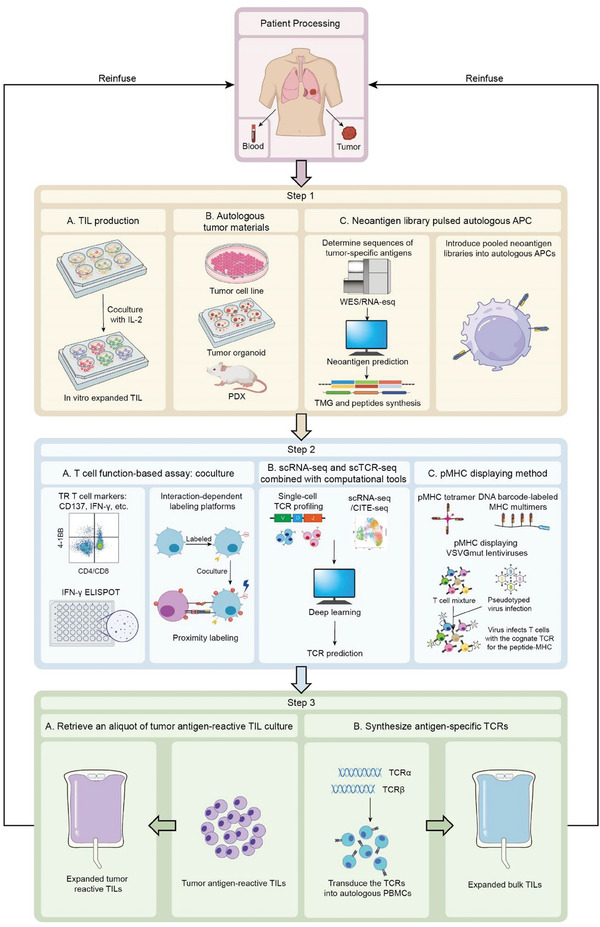
Approaches for Identifying Tumor‐reactive TILs and Their Application in Personalized Immunotherapy. Tumor samples are cultured to generate TILs and construct tumor antigen providers, which include tumor cells, tumor organoids, or PDX models derived from freshly dissected tumors. Additionally, neoantigen library‐pulsed autologous APCs are established. Typically, tumor (T) and normal (N) samples undergo whole exome sequencing (WES) and RNA‐seq and/or immunopeptidome analysis to identify tumor‐specific nonsynonymous mutations. Sequences of candidate antigens are synthesized into peptides and tandem mini‐genes (TMG) screening libraries, which are introduced to autologous APCs. Once TILs and tumor antigen‐presenting cells are prepared, the next step is to identify the TILs that recognize tumor antigens. T cell function‐based assays are commonly used for this purpose. After co‐culturing TILs overnight with APCs, methods such as measuring IFN‐γ production or upregulation of T cell surface activation molecules are employed to identify TILs that recognize tumor neoantigens. Recent advancements in proximity labeling techniques have further enhanced the identification of TILs that target tumor cells. Single‐cell sequencing technologies, combined with previous sequencing datasets, help to construct signatures or algorithms for predicting tumor‐reactive TCRs. Peptide‐major histocompatibility complex (pMHC) display methods, including pMHC‐displaying VSVGmut lentiviruses and DNA barcode‐labeled MHC multimers, provide new strategies for identifying tumor‐reactive TCRs. Finally, tumor‐reactive TILs are expanded to large numbers ex vivo and reinfused into the patients. Alternatively, TCRs that confer tumor antigen recognition can be retrovirally transduced into autologous PBMCs, which are then expanded and reinfused into patients. Both approaches require pretreating the patients with lymphodepleting chemotherapy to enable engraftment of transferred lymphocytes, followed by intravenous IL‐2 administration to stimulate their survival and proliferation.

The following summarizes current efforts in detecting tumor antigen‐specific TILs, including the culture of autologous tumors, the prediction of potential tumor antigens, as well as the methods developed to select tumor antigen‐reactive TILs and the identification of their signatures (**Figure** **2**).

### 7.1 Culture of Autologous Tumor Cells with Tumor Antigenic Profiles

In most cases, we do not know the antigenic epitopes specifically recognized by tumor‐reactive T cells initially. Utilizing autologous tumor materials as tumor antigen presenters is the most direct approach, encompassing tumor single cell suspension, patient‐derived xenografts (PDX), autologous tumor cell lines, and patient‐derived tumor organoids.

The PDX models are established by grafting freshly resected human cancer tissues into immune‐deficient mice. These models preserve genetic profiles and intratumor heterogeneity.^[^ ^69^ ^]^ In the screening of tumor‐reactive T cells, utilizing established PDX models by digesting them into single cell suspensions and co‐culturing with T cells is an effective method that simulates the tumor antigen pool acquired by T cells.^[^ ^70^ ^]^ However, the generation of PDX mouse models is time‐intensive and expensive. The patient's autologous tumor line would be another choice. In the screening and identification of tumor‐reactive TILs and TCRs, autologous tumor cell lines have played a crucial role, especially in melanoma.^[^ ^45^, ^71^ ^]^ However, traditional techniques for culturing primary tumor cell lines are largely ineffective and typically require many months of effort. Patient‐derived tumor organoids (PDTOs) have started to fill the gap in in vitro personalized tumor modeling. These 3D tumor cell lines are developed from surgically dissected tumors.^[^ ^72^ ^]^ They grow in a matrigel‐rich culture medium, which has been optimized to facilitate the prolonged expansion of cancer cells. Organoids recapitulate both the genomic and histopathological characteristics of the source tumors, including copy number alterations, mutation burdens, and individual cancer gene mutations.^[^ ^73^ ^]^ They have proven successful in pharmacological screening. Recently, several groups have successfully identified and defined tumor antigen‐specific T cells utilizing PDTOs in many cancers including melanoma,^[^ ^74^ ^]^ colorectal cancer,^[^ ^75^ ^]^ pancreatic cancer,^[^ ^76^, ^77^ ^]^ cervical cancer,^[^ ^78^ ^]^ liver cancer,^[^ ^79^ ^]^ and lung cancer.^[^ ^80^ ^]^

### 7.2 Identification of Tumor‐Specific Antigens

Although PDX, tumor organoids, and cancer cell lines can authentically represent tumor antigen scenarios, their establishment is time‐consuming, with a low success rate and sometimes requiring large amounts of samples. Consequently, their practical applications are somewhat constrained. Steven Rosenberg et al. employed next‐generation sequencing (NGS) technology to devise a novel screening strategy for the selection of tumor antigen‐specific T cells through computational prediction of tumor antigens.^[^ ^44^, ^81^, ^82^ ^]^ With this strategy, a comparative assessment of WES data from paired tumor‐normal tissues was conducted to identify mutated proteins. The expression levels of mutated genes in tumor tissues were determined through RNAseq, followed by the identification of presumed T cell epitopes based on MHC‐peptide binding prediction algorithms. Subsequently, synthesized antigen peptides were loaded onto autologous APCs to assess the recognition of TILs. Additionally, they proposed a method utilizing a serial of TMG, where these mini‐genes encode peptides with mutated amino acid residues, consisting of 12–13 amino acids with N‐terminal and C‐terminal flanking regions. By synthesizing mini‐genes and transfecting them into APCs, they successfully identified novel antigens in mouse tumor models, as well as in cancer patients, and screened tumor antigen reactive T cells.

### 7.3 Strategies to Screen Tumor‐Reactive TILs

#### 7.3.1 T cell Functionality as the Read‐Outs

Leveraging T cell specificity typically relies on the read‐outs of T cell functionality, specifically referring to cytokine release (such as IFN‐γ, TNF‐α), activation marker upregulation (e.g., CD137, CD134, CD69), cytotoxicity, and clonotype expansion in the presence of candidate antigens. In co‐culture with cancer cells or APCs presenting tumor antigens, T cells produce various cytokines which can be tested by ELISpot, ELISA, or polyfunctional intracellular cytokine staining. Similarly, CD137, as a surface marker of short‐term TCR engagement, can be detected using flow cytometry. A recent study indicates that CD137 upregulation, indicative of T cell reactivity, does not entirely correlate with IFN‐γ secretion.^[^ ^83^ ^]^ This may be due to the TIL dysfunction (exhaustion, anergy, or senescence) with impaired cytokine secretion but still responsive to TCR engagement by upregulating CD137 expression. Nonetheless, clonotype expansion traced by TCR sequencing can be used as a more reliable method since it's the reaction of all T cells upon identifying their specific antigen.^[^ ^84^, ^85^ ^]^

#### 7.3.2 pMHC Multimers to Identify Cancer‐Reactive T Cells

pMHC multimers are commonly employed to detect T cells capable of recognizing tumor‐specific antigens. In the absence of co‐stimulation, the affinity between TCR and pMHC (1–100 µm) is exceedingly weak, resulting in rapid dissociation and difficulty in detecting the interaction between TCR and pMHC.^[^ ^86^ ^]^ Mark M. Davis et al. pioneered the development of pMHC tetramers based on avidin‐biotin interactions, facilitating stable interactions between TCR and pMHC.^[^ ^87^ ^]^ The formation of pMHC multimers involves linking labeled main chains to individual pMHC monomers. Classical methods for staining pMHC multimers include coupling them with fluorescent labels and coculturing them with T cells, allowing the identification of T cells recognizing pMHC multimers through traditional flow cytometry. This method is most suitable for the targeted detection of T cells recognizing common, well‐defined antigens known to bind to HLA. To identify TCRs specific to conserved, immunogenic, and therapeutically actionable epitopes in hotspot mutants, tetramers or even multimers of pMHC, are used to specifically examine TIL responsive to these “public” mutations such as R175H, R248W, and Y220C in *P53*, E545K, E542K and H1047L in *PIK3CA*.^[^ ^70^, ^88^ ^]^ Building on the prediction of candidate HLA‐binding peptides tailored to patients' HLA alleles, the use of heavy metal tags and mass cytometry aids in enhancing the specificity of parallel detection (>100). Similar high‐throughput methods involve DNA barcode labeling of pMHC multimers, allowing for the multi‐parallel screening of over 1000 pMHC molecules though multimers tagged with DNA barcodes.^[^ ^89^ ^]^

#### 7.3.3 Computational Tools

The development of NGS technology has greatly propelled our understanding of the TCR repertoire. Experimental validation of antigen‐specific TCR sequences has opened up new possibilities for predicting T‐cell specificity. Several teams have assembled databases to serve as centralized repositories for information of TCR‐pMHC pairs, such as VDJdb,^[^ ^90^ ^]^ McPAS,^[^ ^91^ ^]^ TCRdb,^[^ ^92^ ^]^ and NeoTCR.^[^ ^93^ ^]^ NeoTCR, for instance, aggregates newly reported and validated antigen‐specific TCR information from the literature, including details on newly identified antigen epitopes, associated gene mutations, HLA restrictions, etc., aiding in the accelerated identification of novel antigen‐specific TCRs from raw sequencing data. Furthermore, numerous groups have developed powerful computational tools, predominantly based on machine learning algorithms, aiming at elucidating the antigen specificity of TCRs,^[^ ^94^ ^]^ such as TCRdist,^[^ ^95^ ^]^ ALICE,^[^ ^96^ ^]^ iSMART,^[^ ^97^ ^]^ GLIPH2^[^ ^98^ ^]^ and netTCR2.0.^[^ ^99^ ^]^ These methods typically input the CDR3 sequences from both TCR α and TCR β chains, clustering them based on the similarities. Matching these sequences with recorded ones allows for inferring T cell specificity. Single‐cell TCR sequencing (scTCR‐Seq) has achieved accurate analysis of TCR α–β pairing, further facilitating experimental endeavors to map TCR repertoires for neoantigens.^[^ ^100^, ^101^, ^102^, ^103^, ^104^ ^]^ With the development and iteration of machine learning methods, incorporating more verified complete TCR‐pMHC pairs as training data, accurate prediction of novel antigen‐specific TCRs may become feasible in the future.

#### 7.3.4 New Technology

Several interaction‐dependent proximity labeling platforms have been reported for detecting antigen‐reactive T cells. The principle involves the transfer of labels from donor cells to recipient cells when the TCR binds to pMHC. Peng Wu et al. established a method termed FucoID which utilizes the interaction‐dependent fucosyl‐biotinylation for identifying antigen‐specific T cells.^[^ ^105^ ^]^ They applied this technique to isolate tumor antigen‐specific T cells from tumor digests, even without any prior information on the specific tumor‐associated antigen identities. Gabriel D. Victora et al. employ bacterial sortase A‐mediated cell labeling to detect receptor‐ligand interaction between immune cells, including TCR‐pMHC interaction. This method is referred to as LIPSTIC.^[^ ^106^, ^107^ ^]^ Several methods, such as EXCELL, PhoXCELL, are based on an evolved variant of sortase A (mgSrtA) to label diverse cell surface proteins with a monoglycine residue at the N‐terminus.^[^ ^108^, ^109^ ^]^

The advent of technologies such as V‐CARMA,^[^ ^110^ ^]^ ENTER,^[^ ^111^ ^]^ RAPTR^[^ ^112^ ^]^ based on lentiviral transfection assays, provides opportunities to screen specific TCRs targeting various displayed pMHC in a library versus library way. Basically, these VSVGmut pseudotyping system achieves specific targeting of TCR‐T cells through pMHC displaying VSVGmut lentiviruses, which requires previous knowledge of the pMHC complexes of interest. Yet the transfection efficiency of primary T cells is low, which may hinder their further application.

### 7.4 Markers of Tumor‐Specific TILs

Recent transcriptomic analysis at the single‐cell level for TILs has revealed common patterns of anti‐tumor T cell functional impairment across cancers and shown the potential to identify anti‐tumor TCRs from TIL transcriptomic and phenotypic profiles. Tumor‐reactive TILs exhibited high PD‐1 expression, and those PD‐1+ TILs showed greater cytotoxicity than PD‐1‐ TILs.^[^ ^71^, ^113^ ^]^ CD39 is another highly expressed marker on empirically defined tumor‐specific CD8 and CD4 T cells in many solid tumors.^[^ ^102^, ^114^, ^115^, ^116^, ^117^, ^118^ ^]^ Also, the CD8+CD69+CD103+ tissue‐resident memory T cell subset is associated with improved efficacy in breast cancer patients receiving checkpoint therapy.^[^ ^119^ ^]^ Furthermore, many studies emphasize the transcriptional expression of *CXCL13*, along with a few other markers, serves as a distinguishing feature of tumor antigen‐specific T cells across various cancers.^[^ ^101^, ^102^, ^103^, ^104^, ^120^, ^121^ ^]^ The expression of *CXCL13* functions as an independent pan‐cancer prognostic indicator of a favorable response to ICB immunotherapy.^[^ ^122^ ^]^ In the “predicTCR50” constructed by Edward W Green et al. based on the TCR reactivity dataset, *CXCL13* emerges as the most significant contributor to the classifier for accurate prediction.^[^ ^123^ ^]^

## 8 Challenges of TIL Therapy for Lung Cancer

As a form of cellular therapy, TIL therapy is logistically much more challenging compared to off‐the‐shelf drugs, especially in reproducibility and scalability. TILs are derived from tumor tissues rather than blood, which presents additional manufacturing challenges. Due to tumor heterogeneity, the process of culturing TIL from tumors lacks strict scientific standards, thereby leading to difficulties in quality control. In addition, TIL manufacturing success rates range from 80% to above 90%, with risks of treatment cancellation due to inadequate TIL production.^[^ ^124^ ^]^ Moreover, the production cycle may exceed one month, posing challenges for rapidly progressing patients. Specialized GMP facilities and trained personnel are required, demanding substantial investment, which in turn, comes with relatively high costs.

As for therapeutic efficacy, in addition to the above‐mentioned parameters of the TIL products, another critical factor affecting efficacy is the ability to homing to tumor sites, which may be largely impaired by abnormal tumor vasculature, deregulated chemokine expression, and complex immunosuppressive microenvironment. Besides, immune suppressive signals and chronic engagement of TCR within the tumor microenvironment can lead to T cell exhaustion and dysfunction, further limiting the persistence and efficacy of T cells.

Adverse effects of TIL therapy necessitate extended hospitalization and may lead to various treatment‐related adverse events (TRAEs), including fever, hypotension, hypoxia, diarrhea, and bone marrow suppression.^[^ ^125^ ^]^ While most toxicities are manageable, some patients may experience persistent autoimmune effects. In a melanoma case, severe autoimmune reactions such as rash, panuveitis, and hearing loss occurred post‐TIL therapy.^[^ ^126^ ^]^ Elevated levels of MART‐1 MHC multimer‐positive CD8+ cells were detected in ocular biopsies post‐treatment compared to peripheral blood. The patient achieved complete remission after 2 years, suggesting a potential positive correlation between TIL therapy‐induced immune‐related adverse events (irAEs) and treatment response. This parallels the relationship observed between ICB‐induced irAEs and efficacy, yet further confirmation in larger cohorts is needed.^[^ ^127^ ^]^ Additionally, NSCLC directly affects pulmonary function, with individuals having smoking‐related NSCLC exhibiting a high prevalence of pulmonary and cardiac comorbidities. In phase I clinical trial (NCT03215810), two NSCLC patients with compromised cardiopulmonary function experienced severe TRAEs of Grade>3 and succumbed before response evaluation.^[^ ^52^ ^]^ Similarly, in the phase II multicenter study (NCT03645928) of lifileucel in ICI‐treated metastatic NSCLC patients, a 60‐year‐old woman died of heart failure.^[^ ^54^ ^]^ Their treatment toxicity resembles typical toxicities observed with lymphodepletion chemotherapy and high‐doseIL‐2, underscoring the critical importance of meticulous patient selection, particularly regarding cardiopulmonary reserve, for the success of this therapy.

## 9 Next generation of TIL Therapy

Future research and political prioritization are needed to expand access to TIL therapy, ensuring its availability to a broader spectrum of patients (as reviewed by Tine J Monberg et al.^[^ ^125^ ^]^). Here we mainly focus on the strategies to improve treatment efficacy and decrease toxicity.

### 9.1 Improving TIL Efficacy

Substantial efforts are being made to identify the optimal T cell populations, and the selection markers to predict the enhanced tumor‐specific responses. Currently, there is no clear consensus on how to isolate the most effective TIL subsets, the subsequent process generally involves selecting TILs or transducing primary T cells with tumor‐specific TCRs, followed by expansion (Figure 2). TIL therapy was also found to correlate patient response with a less differentiated phenotype and prolonged persistence, which is closely associated with maintaining TlL clone diversity and avoiding dysfunctional phenotypes.

Improving culture conditions to reprogram T cell metabolism is a straightforward approach. Activation of the PI3K/AKT signaling pathway boosts T cell glycolysis, growth, and effector functions. Multiple studies have shown that inhibiting AKT pharmacologically induces a phenotype with early memory in TILs and CAR‐T cells.^[^ ^128^, ^129^, ^130^, ^131^ ^]^ Another simple option is to add optimized cytokines. T cells cultured with IL‐15 and IL‐7 have been shown to exhibit enhanced long‐term persistence compared to those cultured with IL‐2 and IL‐7. This improvement is attributed to their enhanced metabolic fitness.^[^ ^132^, ^133^ ^]^ In the culture medium, [K+] serves as a regulator of T cell stemness. Suman Kumar Vodnala et al. discovered that elevated [K+] elicited a T cell starvation reaction, subsequently inducing autophagy. This process drove a metabolic and epigenetic shift in T cells, thereby restricting the acquisition of effector functions while maintaining TCF7 expression and functional stem‐like properties.^[^ ^134^ ^]^ Recently, Jan P. Böttcher et al.^[^ ^135^ ^]^ and George Coukos et al.^[^ ^136^ ^]^ respectively show that PGE2 prevents TIL growth by disrupting IL‐2 signaling and mitochondrial function. Blockade of the PGE2–EP2/EP4 axis using COX inhibitor ketorolac during the culture process boosts TIL proliferation, fitness, and anti‐tumor activity. These methods offer feasible, reversible, and convenient in vitro approaches to enhance T cell function albeit the effects seem temporary.

Enhancing TIL potency can be achieved through gene modification techniques, especially CRISPR editing, in a permanent manner. Studies have been focused on knocking out inhibitory molecules that induce T cell exhaustion and poor proliferation, such as PD‐1^[^ ^137^ ^]^ and PRDM1.^[^ ^138^, ^139^ ^]^ A phase I/II trial (NCT05361174) is underway to investigate PD1‐deficient TILs in patients with advanced NSCLC or melanoma. The potential of incorporating proliferation‐enhancing cytokines into TIL products is also under study. CytoTIL15 is a TIL product genetically modified to express a regulated variant of membrane‐bound IL15 (mbIL‐15), which is controlled by carbonic anhydrase‐2 drug‐responsive domains and activated by acetazolamide.^[^ ^140^ ^]^ T cells cultured with IL‐15^[^ ^141^ ^]^ or anchored with IL‐12^[^ ^142^ ^]^ are also under investigation. Furthermore, cytokine‐induced SH2 protein, CISH, suppresses CD8+ T cell signaling. CISH knockout results in enhanced tumor reactivity and cytokine production in TILs and is currently undergoing evaluation in two clinical trials (NCT05566223 and NCT04426669).^[^ ^143^ ^]^ The gene fusion of CARD11‐PIK3R3, discovered in cutaneous T cell lymphoma, is reported to amplify CARD11‐BCL10‐MALT1 complex signaling and increase the anti‐tumor potential of T cells. Precise genomic incorporation of CARD11‐PIK3R3 into TILs is a promising way for potency enhancement.^[^ ^144^ ^]^ Recently, Evan W. Weber et al.^[^ ^128^ ^]^ and Phillip K. Darcy et al.^[^ ^132^ ^]^ independently identified FOXO1 as a master regulator of memory programming in both modified and unmodified T cells. T cells overexpressing FOXO1 exhibit gene expression profiles akin to that of stem cells, along with a rejuvenated ability to combat cancer.

Furthermore, next‐generation TIL therapy can be optimized in combination with standard cancer treatments such as chemotherapy, radiotherapy, and ICB therapy for lung cancer. Alternatively, a prime‐boost regimen involving prior vaccination could first activate tumor‐specific T cells, followed by TIL therapy to synergistically augment anti‐tumor immune responses.^[^ ^145^, ^146^ ^]^ Moreover, vaccination might serve as a booster post‐ACT to facilitate effective re‐stimulation and infiltration of infused TILs in vivo.^[^ ^147^ ^]^ Overall, the initiation of appropriate combination approaches is forecasted to yield significant enhancements in clinical outcomes.

### 9.2 Reducing TIL Toxicity

Most toxicities associated with TIL therapy emerge from the administration of non‐myeloablative lymphodepleting chemotherapy and high dose IL‐2.^[^ ^148^, ^149^, ^150^ ^]^ To mitigate toxicity derived from lymphodepleting chemotherapy, alternative regimens including dose reduction of cyclophosphamide or the duration shortening of chemotherapy are undergoing testing.^[^ ^151^ ^]^ In addition, high‐dose IL‐2‐caused toxicity could be lessened via the replacement with IL‐2 variants with higher affinities for the IL‐2Rβγ expressed on limited cells instead of conventional IL‐2 with high‐affinity for IL‐2Rαβγ widely expressed on vascular endothelium. Such engineered IL‐2 with enhanced therapeutic potential and lower toxicity like Neoleukin 2/15,^[^ ^152^ ^]^ Orthogonal IL‐2^[^ ^153^ ^]^ and H9T^[^ ^154^ ^]^ are developed or under clinical evaluation. Directing cytokines exclusively to TILs could offer a method of distinguishing the beneficial impacts of these agents on TILs from the side effects they provoke when administered systemically. Wendell A. Lim et al. design the synthetic Notch (synNotch) receptor and use it to create synthetic IL‐2 circuits in engineered‐T cells. This establishes a signaling bypass mechanism that induces the production of local IL‐2 upon synNotch's recognition of a tumor. This synthetic IL‐2 pathway induces potent TCR‐T or CAR‐T cell infiltration and effective tumor regression in immune‐excluded solid tumor models while avoiding associated systemic or off‐target toxicities.^[^ ^155^ ^]^

## 10 Conclusion

Clinical trial data from recent years have demonstrated a considerable efficacy of TIL therapy in advanced NSCLC, with effectiveness also seen in patients previously treated with ICI. Therefore, TIL therapy offers a meaningful complement to lung cancer treatment strategies and holds promise for advancing the field of adoptive cell therapy for lung cancer. Excitingly, advancements in tumor‐recognizing T cell selection strategies and cell engineering present promising and rapidly evolving possibilities to further optimize TIL therapy for patients with solid tumors. However, the increased complexity in the manufacturing process may prolong the production timeline and increase the risk of disease progression or patient dropout before treatment initiation. Thus, future research needs to carefully assess the balance between patient outcomes and cost‐effectiveness. Nevertheless, we believe that with a deepened understanding of the tumor microenvironment as well as advanced technology development, there will be more opportunities to expand the therapeutic window and unlock the true potential of TIL therapy for lung cancer treatment.

## Conflict of Interest

The authors declare no conflict of interest.

## Author Contributions

W.H. and Y.B. contributed equally to this work. Conceptualization was done by H.J. and W.H. Investigation was done by W.H., Y.B., and H.J. Original Draft was written by W.H. and Y.B. H.J. dealt with Writing the Review and Editing. Funding Acquisition was done by H.J. and W.H. Supervision was done by H.J. All authors revised and approved the final version of the manuscript.
